# N-Glycoside of Indolo[2,3-*a*]pyrrolo[3,4-*c*]carbazole LCS1269 Exerts Anti-Glioblastoma Effects by G2 Cell Cycle Arrest and CDK1 Activity Modulation: Molecular Docking Studies, Biological Investigations, and ADMET Prediction

**DOI:** 10.3390/ph17121642

**Published:** 2024-12-06

**Authors:** Nikolay Kalitin, Natalia Koroleva, Anna Lushnikova, Maria Babaeva, Nadezhda Samoylenkova, Ekaterina Savchenko, Galina Smirnova, Yulia Borisova, Alexander Kostarev, Aida Karamysheva, Galina Pavlova

**Affiliations:** 1 Laboratory of Tumor Cell Genetics, N.N. Blokhin National Medical Research Center of Oncology, Kashirskoe Shosse 24, 115478 Moscow, Russia; aikaram@yandex.ru; 2 Laboratory of Oncogenomics, N.N. Blokhin National Medical Research Center of Oncology, 115478 Moscow, Russia; nat.korole@yandex.ru (N.K.); lan21@yandex.ru (A.L.); 3 Molecular Medicine, Universitätsmedizin Berlin, 10117 Berlin, Germany; marybabaeva@mail.ru; 4 Laboratory of Molecular and Cellular Neurogenetics, N.N. Burdenko National Medical Research Center of Neurosurgery, 125047 Moscow, Russia; samoylenkova.n@gmail.com (N.S.); savhenko61@mail.ru (E.S.); lkorochkin@mail.ru (G.P.); 5 Laboratory of Biochemical Pharmacology and Tumor Models, N.N. Blokhin National Medical Research Center of Oncology, 115478 Moscow, Russia; gsmir53@yandex.ru (G.S.); ulkabor@yandex.ru (Y.B.); 6 Max Planck Institute for Biology, University of Tübingen, 72074 Tübingen, Germany; aleksandrkostarevv@gmail.com; 7 Laboratory of Neurogenetics and Developmental Genetics, Institute of Higher Nervous Activity and Neurophysiology of RAS, 117485 Moscow, Russia

**Keywords:** LCS1269, glycosylated indolocarbazoles, CDK1, Wee1, Myt1, glioblastoma

## Abstract

**Background/Objectives:** Indolo[2,3-*a*]pyrrolo[3,4-*c*]carbazole scaffold is successfully used as an efficient structural motif for the design and development of different antitumor agents. In this study, we investigated the anti-glioblastoma therapeutic potential of glycosylated indolocarbazole analog LCS1269 utilizing in vitro, in vivo, and in silico approaches. **Methods:** Cell viability was estimated by an MTT assay. The distribution of cell cycle phases was monitored using flow cytometry. Mitotic figures were visualized by fluorescence microscopy. Quantitative RT-PCR was used to evaluate the gene expression. The protein expression was assessed by Western blotting. Molecular docking and computational ADMET were approved for the probable protein target simulations and predicted pharmacological assessments, respectively. **Results:** Our findings clearly suggest that LCS1269 displayed a significant cytotoxic effect against diverse glioblastoma cell lines and patient-derived glioblastoma cultures as well as strongly suppressed xenograft growth in nude mice. LCS1269 exhibited more potent anti-proliferative activity toward glioblastoma cell lines and patient-derived glioblastoma cultures compared to conventional drug temozolomide. We further demonstrated that LCS1269 treatment caused the severe G2 phase arrest of cell cycle in a dose-dependent manner. Mechanistically, we proposed that LCS1269 could affect the CDK1 activity both by targeting active site of this enzyme and indirectly, in particular through the modulation of the Wee1/Myt1 and FOXM1/Plk1 signaling pathways, and via p21 up-regulation. LCS1269 also showed favorable pharmacological characteristics in in silico ADME prediction in comparison with staurosporine, rebeccamycin, and becatecarin as reference drugs. **Conclusions:** Further investigations of LCS1269 as an anti-glioblastoma medicinal agent could be very promising.

## 1. Introduction

Glioblastoma multiforme (GBM), also referred as a grade IV astrocytoma, is the most common and aggressive malignant brain tumor in adults, causing 3–4% of all cancer-related deaths [1,2]. Despite the progress made in the current standard of high-intensity treatment regimen including surgery as safely feasible followed by radiotherapy plus concomitant and maintenance temozolomide pharmacotherapy, the outcome for GBM patients remains extremely poor with a median overall survival less than 20 months [3,4,5]. Currently, the drug resistance to temozolomide is the most intractable problem in GBM management; therefore, the new drugs extremely need to be developed for GBM combined therapy [6].

Various kinases and multiple signaling pathways could be potential therapeutic targets due to their abnormal activation in fast-growing glioblastoma cells [7,8,9,10]. In particular, drugs targeting cyclin-dependent kinases (CDKs), enzymes controlling the cell cycle progression in both normal and tumor cells, are widely used in the treatment of human solid and hematological malignancies [11]. Seliciclib (roscovitine or CYC202), a first-generation pan-CDK inhibitor with preferentially inhibitory activities toward CDK2, CDK7, and CDK9, is not approved nor widely used in clinical practice because of the toxic effect in GBM patients, while it exhibited antitumor activity on GBM patient-derived xenografts and glioblastoma cell lines [12,13]. To date, only three FDA (U.S. Food and Drug Administration)-approved second-generation inhibitors specifically targeted CDK4 and CDK6, abemaciclib, palbociclib, and ribociclib, are the front-line treatment for the advanced breast cancer [14,15]. As for GBM, a few studies have investigated the therapeutic potential of CDK4/6 inhibitors and the results obtained are controversial [16,17,18]. Even though at present there are approximately 40 pan-CDK inhibitors, only a few drugs, such as dinaciclib and indirubin, exert broad-spectrum pan-CDK inhibitory potential, promising antitumor activity in both solid and non-solid tumors, and minor side effects [19].

The indolo[2,3-*a*]pyrrolo[3,4-*c*]carbazoles isomers belong to the class of hexacyclic fused carbazoles structurally characterized by an indole moiety coupled to one of the benzene rings of a carbazole skeleton [20]. Such substances often occur naturally as well as being produced synthetically and by means of combinatorial biosynthesis [21]. Thus, indolopyrrolocarbazoles of natural origin were found in a number of bacteria, blue-green algae, and a few tunicates, mollusks, and sponges [21,22]. The antibiotic staurosporine is the first indolo[2,3-*a*]carbazole derivative of natural origin isolated from a culture of *Streptomyces staurosporeus* and exhibited the diverse biological potencies, such as the inhibition of protein kinase C, antimicrobial, anti-parasitic activities, and antitumor properties [23,24].

Over the last 40 years, a number of indolo[2,3-a]carbazoles and staurosporine analogs, such as synthetic compounds K-252a and UCN-01, have been synthesized and tested as potent anticancer agents [25,26]. Molecular structures of both natural and synthetic alkaloids belonging to indolopyrrolocarbazole family commonly constitute a “close” indolo[2,3-*a*]pyrrolo[3,4-*c*]carbazole scaffold which is joined to a carbohydrate fragment, so that the carbohydrate moiety can be linked to the indolopyrrolocarbazole skeleton by means of one (for example, in rebeccamycin) or two (for example, in staurosporine) N-glycosidic bonds [27]. Notably, the indolo[2,3-*a*]pyrrolo[3,4-*c*]carbazole analogs can trigger the DNA damage features, affect the activity of CDK2, Akt1, Erk1/2, and ribosomal S6 kinases, modulate the topoisomerase I activity, as well as modify intracellular signaling pathways, such as p53, EGFR, mTOR, NF-kB, and JAK/STAT [28,29,30,31,32]. It had also been noted that sugar fragments tethered with indolopyrrolocarbazole backbones increase the efficiency of these compounds binding with their targets in contrast to indolopyrrolocarbazoles lacking the ones [33].

Previously, we and our colleagues have studied the antitumor potential of LCS1269 selected from a set of previously synthesized N-glycosylated indolocarbazole derivatives as a lead compound [34,35]. Moreover, it was established that LCS1269 could overcome the multidrug resistance of resistant melanoma cells to DNA-damaging drugs [36]. Another study identified some molecular mechanisms of LCS1269-mediated anticancer activity [37].

In summary, the rational design and synthesis of new glycosylated indolo[2,3-*a*]pyrrolo[3,4-*c*]carbazole derivatives characterized by a broad spectrum of robust antitumor activities in respect of the key intracellular oncogenic targets may constitute a promising strategy for anticancer therapy.

## 2. Results and Discussion

### 2.1. LCS1269 Reduces Proliferation of Both Glioblastoma Cell Lines and Patient-Derived Glioblastoma Cell Cultures

**Figure 1:**
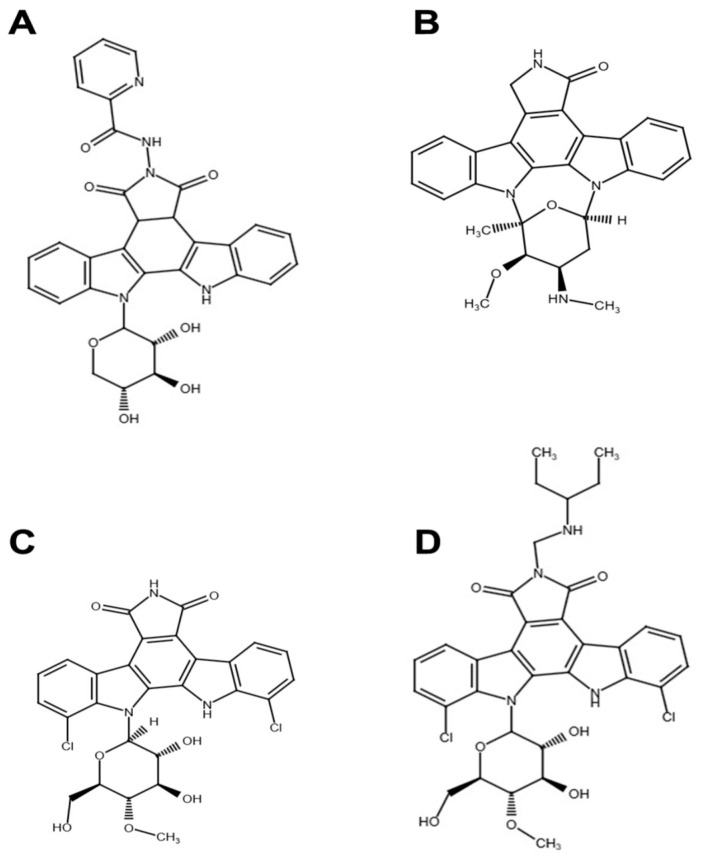
Molecular structures of (**A**) LCS1269, (**B**) Staurosporine, (**C**) Rebeccamycin, and (**D**) Becatecarin.

We have previously reported the general synthetic route for preparation of N-glycosides of indolo[2,3-a]pyrrolo[3,4-c]carbazole that demonstrated the robust growth inhibitory activity against different mice transferable solid tumors and were generated in good yields under the condition of simple workup and purification [34]. Among synthesized compounds, 6-picolinamido-12-(β-D-xylopyranosyl)-indolo[2,3-a]pyrrolo[3,4-c]carbazole-5,7-dione (LCS1269) containing pyridine-2-carboxylic acid (picolinic acid) moiety, possessed the highest potency toward a range of cancer cell lines compared to non-tumor cells [34]. The chemical structure of LCS1269 is presented in Figure 1A.

**Figure 2:**
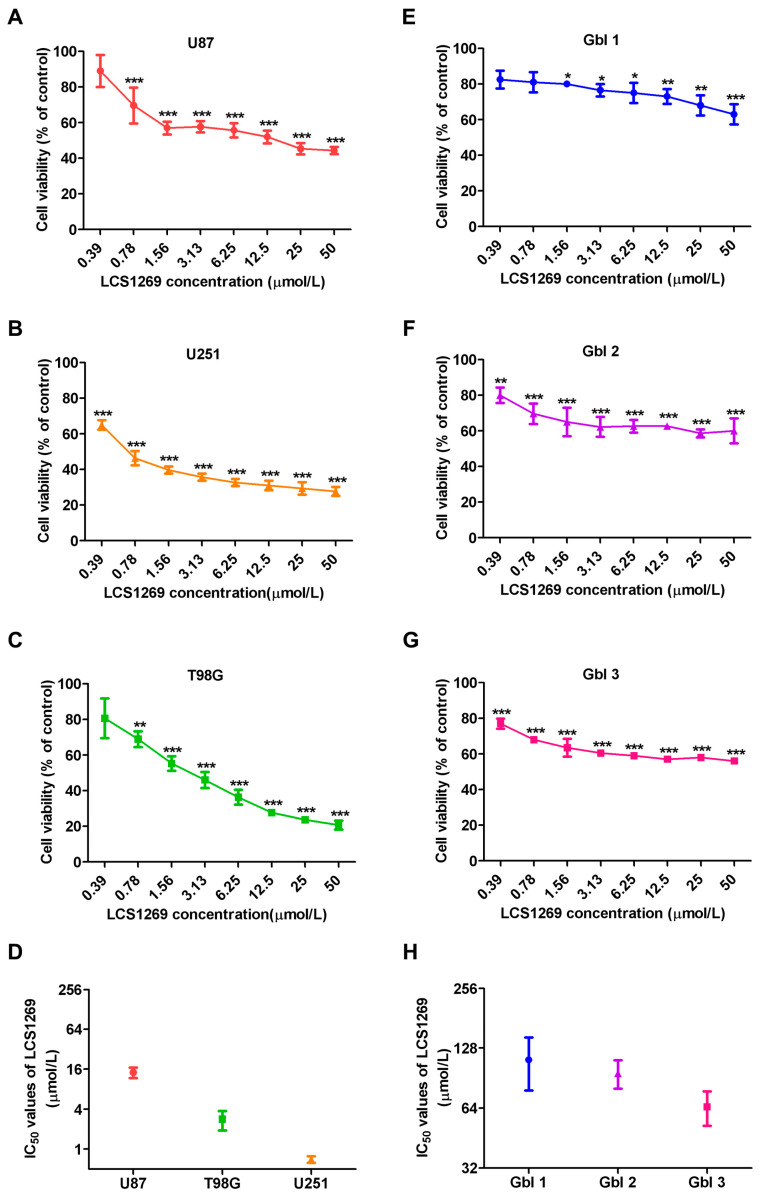
Inhibitory effect of LCS1269 on cell viability of human glioblastoma cell lines and patient-derived glioblastoma cell cultures. (**A**–**C**) The viability of three glioblastoma cell lines treated with the indicated concentrations of LCS1269 for 72 h. (**D**) IC_50_ values of LCS1269 for U87, U251, and T98G cell lines. (**E**–**G**) LCS1269 treatment for 72 h reduces the viability of three patient-derived glioblastoma cell cultures in a dose-dependent manner. (**H**) IC_50_ values of LCS1269 for Gbl1, Gbl2, and Gbl3 cell cultures. Data are presented as mean ± SD. * *p* < 0.05, ** *p* < 0.01, and *** *p* < 0.001 as compared with the untreated cells.

In order to determine if LCS1269 exerts any influence on the viability of human glioblastoma cells, we investigated its cytotoxic activity against three human GBM lines (U87, U251, and T98G) and three patient-derived GBM cell lines (Gbl 1, Gbl 2, and Gbl 3) using standard MTT method. U87 cells with IC_50_ 14.33 ± 2.52 μM were the most resistant to the toxic action of LCS1269, whereas U251 cells with IC_50_ 0.70 ± 0.08 μM were the most susceptible ones (Figure 2A–C).

The resistance of T98 GBM cells to the anti-proliferative activity of LCS1269 was intermediate (IC_50_ 2.83 ± 0.93μM). The IC_50_ of the most sensitive U251 and the most resistant U87 cells differed by more than 10 times (Figure 2D). While the patient-derived GBM cell cultures were more resistant to the growth-inhibitory activity of LCS1269 than GBM cell lines, we revealed that the antiproliferative effect of LCS1269 on the patient-derived GBM cells was dose-dependent and significant (Figure 2E–G). The more resistant patient-derived GBM lines were Gbl 1 (IC_50_ 111.67 ± 33.23 μM) and Gbl2 (IC_50_ 95.67 ± 15.63 μM), whereas Gbl 3 with IC_50_ 65.00 ± 12.77 μM was the most susceptible culture (Figure 2H).

**Figure 3:**
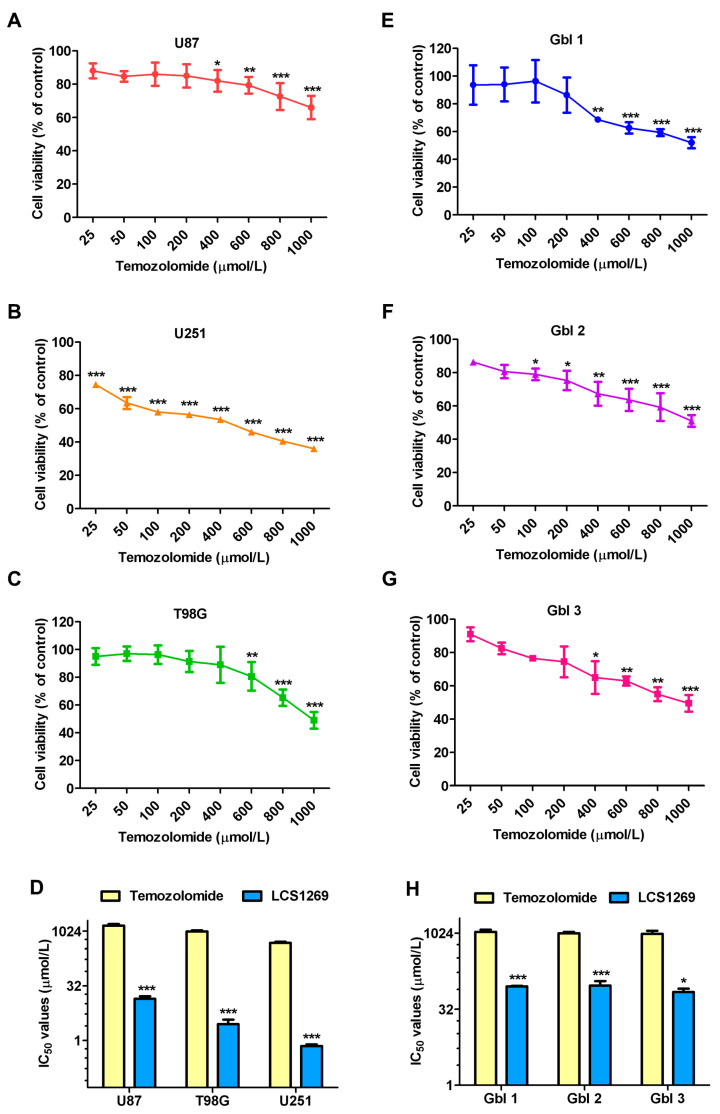
Inhibitory effect of conventional anti-glioblastoma drug temozolomide on cell viability of human glioblastoma cell lines and patient-derived glioblastoma cell cultures and therapeutic potency comparison of temozolomide and LCS1269. (**A**–**C**) The viability of three glioblastoma cell lines treated with the indicated concentrations of temozolomide for 72 h. (**D**) IC_50_ values comparison of temozolomide and LCS1269 for U87, U251, and T98G cell lines. (**E**–**G**) temozolomide treatment for 72 h reduces the viability of three patient-derived glioblastoma cell cultures in a dose-dependent manner. (**H**) IC_50_ values comparison of temozolomide and LCS1269 for Gbl1, Gbl2, and Gbl3 cell cultures. Data are presented as mean ± SD. * *p* < 0.05, ** *p* < 0.01, and *** *p* < 0.001 as compared with the untreated cells.

The LCS1269 toxicity towards glioblastoma cells was further compared with that of temozolomide, the drug which is commonly used in GBM chemotherapy. Both GBM cell lines (Figure 3A–C) and patient-derived GBM cell cultures (Figure 3D–F) were more resistant to the toxic action of temozolomide than to the LCS1269 toxicity, and the difference was statistically significant (Figure 3G,H).

As LCS1269 cytotoxic effects were more pronounced in GBM cell lines, U87, U251, and T98G cells were further used in our experiments to highlight the mechanisms of anti-glioblastoma activity of LCS1269.

### 2.2. LCS1269 Strongly Inhibits Tumorigenesis In Vivo

**Figure 4:**
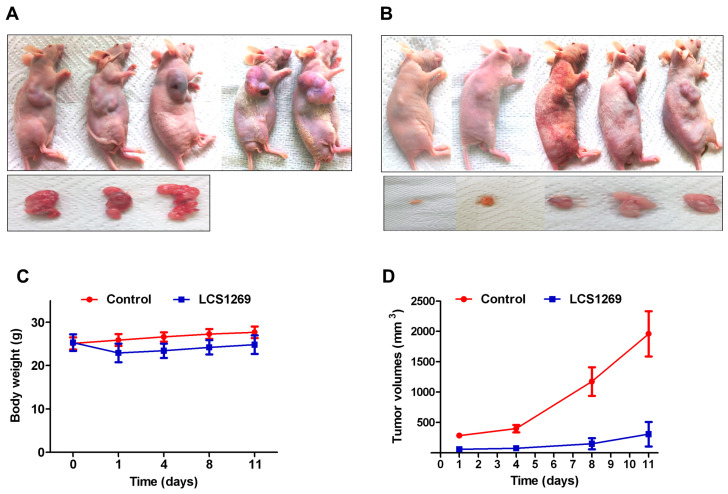
Antitumor efficacy of LCS1269 against xenografts in U87-bearing nude mice (*n* = 5). (**A**) Representative image of xenograft-bearing animals (**upper panel**) and extirpated tumors (**lower panel**) from control group. (**B**) Representative image of xenograft-bearing animals (**upper panel**) and extirpated tumors (**lower panel**) from LCS1269-treated group. (**C**) Body weight changes in nude mice from control and LCS1269-treated groups. (**D**) Changes in U87 xenograft volumes in control and LCS1269-treated groups of nude mice. Data are presented as mean ± SD.

U87 cells were used to investigate LCS1269 effect on xenograft growth delay in immunocompromised mice. The cells were subcutaneously inoculated in five control and five experimental mice treated with LCS1269 (60 mg/kg) 48 h after the U87 inoculation every 24 h for five days. After 11 days of the experiment, all mice were sacrificed, and the tumors of control mice (Figure 4A) and mice treated with LCS1269 (Figure 4B) were isolated.

Although the body weight of the experimental mice was lower than that of the control mice, the difference was not significant (Figure 4C). On the contrary, the tumor volumes of the experimental mice treated with LCS1269 on the 11th day of the experiment was 4–5 times smaller than tumor volumes of the control mice (Figure 4D). These data suggest the ability of LCS1269 to considerably retard the xenograft development in U87-bearing athymic nude mice.

### 2.3. LCS1269 Delays Interphase-to-Mitosis Transition by Inducing G2 Block of Cell Cycle

**Figure 5:**
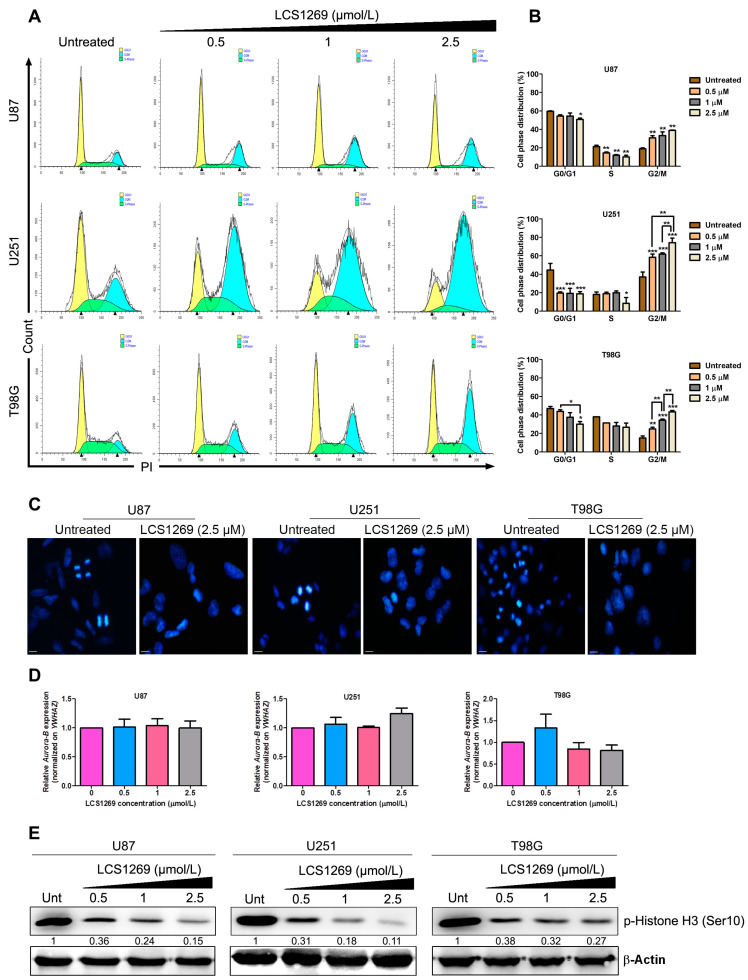
LCS1269 promotes G2 cell cycle block in glioblastoma cell lines. (**A**) Influence of LCS1269 different concentrations treatment (0.5, 1, and 2.5 µM) for 24 h on cell cycle progression using flow cytometry. (**B**) Quantification of cell cycle phase distribution after LCS1269 treatment for 24 h. (**C**) U87, U251, and T98G cells were treated with LCS1269 for 24 h, stained with Hoechst 33258 and visualized using fluorescence microscopy (scale bar = 10 µm). (**D**) Quantitative real-time PCR data of *Aurora-B* gene expression in glioblastoma cell lines treated with LCS1269 in the indicated concentration for 24 h. (**E**) Western blot analysis of p-Histone H3 (Ser10) protein level after LCS1269 treatment for 24 h. β-Actin served as a loading control. Data are presented as mean ± SD. * *p* < 0.05, ** *p* < 0.01, and *** *p* < 0.001 as compared with the untreated cells.

The effect of LCS1269 on the cell cycle progression of GBM cell lines was investigated (Figure 5A,B).

Only the highest concentrations of LCS1269 (2.5 μM) had the arresting effect on G0/G1 phase of cell cycle in U87 and T98G cells (*p* < 0.05), while in U251 cells, the G0/G1 phase was significantly diminished (*p* < 0.001) even at the minimal concentrations (0.5 μM) of LCS1269. No difference in the S phase of the cell cycle was found in T98G cells, and the statistically significant decrease (*p* < 0.05) of the S phase in U251 cells was observed only with the highest concentration of LCS1269. In U87 cells, the dose-dependent decrease in the S phase could be seen (*p* < 0.01). Taken together, the dose-dependent accumulation of glioblastoma cells at G2/M phase under the LCS1269 action was found in all three GBM cell lines.

Since the quantitation of DNA content using propidium iodide and flow cytometry cannot distinguish between G2 and mitotic (M) phase cells, we further used Hoechst 33258 staining combined with mitosis-specific marker (e.g., phospho-histone H3) detection to discriminate the LCS1269 effects on the G2 and M phases separately. To investigate if any mitotic stages could be identified under the fluorescent microscopy in GBM cell lines stained with Hoechst 33258, we were looking for the prophases, metaphase plates, and anaphases/telophases in untreated glioblastoma cells and after the addition of LCS1269. We were able to find the metaphase plates, rare prophases and anaphases/telophases in U87, U251, and T98G glioblastoma cells untreated with LCS1269, but no such kind of structures were observed in any of these cells after the action of LCS1269 (Figure 5C).

One of the major functions in the control of mitotic entry is played by the Aurora kinase family comprising Aurora A, B, and C [38]. Aurora kinases A and B regulate mitosis, while Aurora C regulates meiosis. Aurora B together with the other regulatory subunits (inner centromere protein (INCENP), survivin, and borealin) constitutes the chromosomal passenger complex as a central part in chromosome condensation and movement [39]. Aurora B is the most active during mitosis. In early G2, Aurora B is responsible for chromosome condensation by the phosphorylation of histone H3 on Ser10 initiating the mitotic condensation during G2/M transition [40]. In our study, *Aurora B* mRNA expression was not changed in any of glioblastoma cells after LCS1269 addition (Figure 5D). Nevertheless, the dose-dependent decrease in histone H3 phosphorylation on Ser10 (p-histone H3 (Ser10)) was clearly detected in all three glioblastoma cell lines following the LCS1269 treatment (Figure 5E).

The p-histone H3 (Ser10) down-regulation in spite of the unchanged *Aurora B* expression could be explained by the impairment of Aurora B activity. We tried to check this possibility by means of molecular docking (Supplementary, Figure S1). The simulation of the Aurora B (in complex with INCENP (PDB ID: 4AF3)) interaction with LCS1269 ligand showed a relatively low glide score function equal to −5.20. However, 3D localization and the assumed formation of three stable hydrogen bonds indicate the possibility of these interactions. In particular, some stable hydrogen bonds are predicted: a donor H-bond between Hid250 and the 3′-OH group of carbohydrate moiety and an acceptor H-bond between Asn205 and the carbonyl group in the pyrrole ring. Another donor H-bond between same residue Asn205 and the NH of LCS1269 amide group were simulated. The two cation–π interactions between the indole rings of LCS1269 skeleton and positively charged Lys202 was conceivably observed. Moreover, the pyridine cycle of picolinic acid could form π–π stacking interactions with Phe88. Thus, the molecular docking simulations suggest a feasibility of LCS1269 ligand interaction with Aurora B as a target leading to its functional impairment.

Histone H3 could also be dephosphorylated as a result of the stress response, such as DNA damage and/or oxidative imbalance [41]. The LCS1269 impact on other histone H3 (Ser10) phosphorylating kinases, such as vaccinia-related kinase 1 (VRK1), could also not be excluded [42].

The phosphorylation of Histone H3 on Ser10 is one of the important epigenetic markers of mitosis. The post-translational modifications of histones are implicated in the regulation of a lot of biological processes, including transcription. Such kinds of modifications may, in particular, participate in the silencing of a given gene [43]. The phosphorylation of Histone H3 is of special interest as it was shown that the phosphorylation on Ser10 in the N-terminal tail of Histone H3 is essential for the cell cycle progression and is closely associated with chromosome condensation during mitosis [44]. Histone H3 phosphorylation at Ser10 reaches its maximum level during metaphase, and then decreases rapidly upon transition to anaphase [45]. Overall, p-Histone H3 (Ser10) down-regulation in all three glioblastoma cell lines following LCS1269 treatment indicated the reduction in the mitotic activity in these cells.

### 2.4. LCS1269 May Attenuate CDK1 Activity Both Directly and by Affecting Wee1/Myt1 Signaling

**Figure 6:**
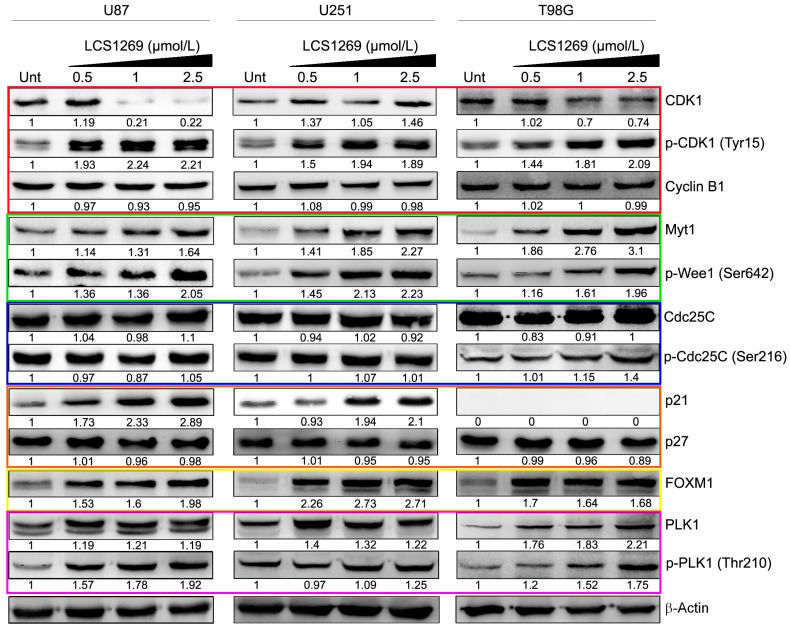
LCS1269 partially affects CDK1 activity by regulation of Wee1/Myt1 and FOXM1/Plk1 signaling pathways, and p21 up-regulation. Western blot analysis of CDK1, p-CDK1, cyclin B1, Myt1, p-Wee1, Cdc25C, p-Cdc25C, p21, p27, FOXM1, Plk1, and p-Plk1 protein levels in U87, U251, and T98G cells treated with LCS1269 in indicated concentrations for 24 h. β-Actin served as a loading control.

The mitotic progression in human cells is highly dependent on the activity of cyclin-dependent kinase 1 (CDK1), one of the key enzymes that control the cell cycle progression and facilitate the cell cycle transition from the G2 phase into mitosis [46]. CDK1 acts in combination with cyclin B1, constituting the M-phase promoting factor (MPF). CDK1-cyclin B1 complexes further migrate to the nuclei to phosphorylate their substrates [47]. While cyclin B1 expression was not changed in any of glioblastoma cell lines after LCS1269 treatment, CDK1 protein expression was decreased in U87 and T98G cells and to a lesser extent in U251 cells (Figure 6, red frame).

The activity of CDK1 is regulated by phosphorylation: dephosphorylation at Tyr15 and Thr14 and phosphorylation at Thr161 activate CDK1-Cyclin B1 complexes, while inhibitory phosphorylation of conserved CDK1 Tyr15 and Thr14 residues provides inactivating effect [47]. LCS1269 addition increased the phosphorylation of Tyr 15 p-CDK1 (Tyr15) (inhibited form) in all glioblastoma cell cultures. The increase in p-CDK1 (Tyr15) could be the result of the diminished dephosphorylating activity or augmentation of Tyr15 phosphorylation of CDK1.

Cell division control protein phosphatase 25C (Cdc25C) dephosphorylates the inactivating phosphorylation of CDK1 on Tyr15, providing the reactivation of this protein kinase followed by mitotic entry [48]. Cdc25C expression was rather high in all the glioblastoma cells studied, and LCS1269 treatment had no appreciable effect on its expression (Figure 6, blue frame). No inhibiting action of LCS1269 on Cdc25C activity was observed as well, as increased concentrations of this substance did not change the expression of p-Cdc25C (Ser 216), the phosphorylated form that prevents functional interactions between Cdc25C and cyclin B1-CDK1 complex. Hence, these data indicate that CDK1 dephosphorylation by Cdc25C triggering mitotic entry is not impaired by LCS1269 action.

The inhibitory phosphorylation of CDK1 is performed by two Wee family kinases, Wee1 and Myt1, with Wee1 exclusively active toward Tyr15 and Myt1, dual-specific to both Tyr15 and Thr14, and predominantly phosphorylating Thr14 [49]. The phosphorylation of the Ser642 residue of Wee1 creates its stabilized active form that in turn phosphorylates CDK1 on the Tyr15, thus preventing G2/M progression [50]. The expression of both Myt1 and Wee1 phosphorylated on Ser642 was dose-dependently activated in glioblastoma cells due to LCS1269 action (Figure 6, green frame), which may result in the augmentation of the inhibited p-CDK1 (Tyr15) maintaining the cyclin B1-CDK1 complex in the inactive state and preventing G2/M progression.

**Figure 7:**
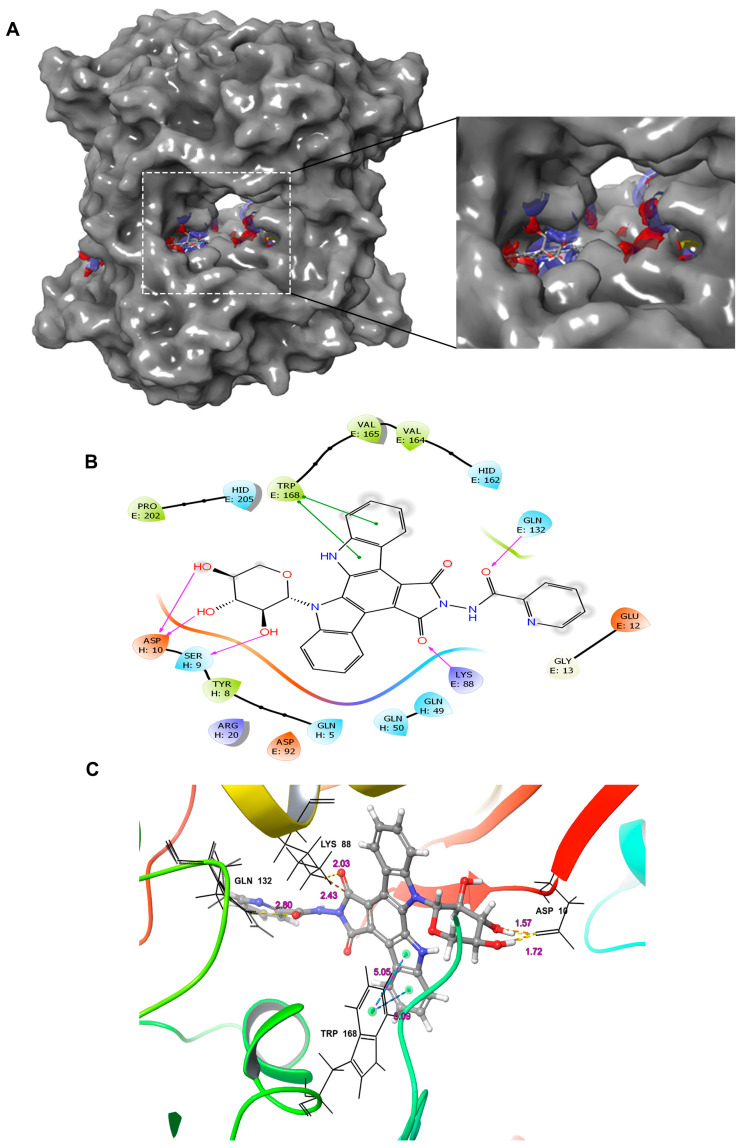
Ligand–protein interaction modeling based on the molecular docking of LCS1269 with CDK1. (**A**) 3D cartoon binding mode, (**B**) 2D binding mode (purple arrows show hydrogen bonds while green lines demonstrate π–π stacking interactions), and (**C**) 3D binding mode (yellow dashed lines denote hydrogen bonds; pink-blue dashed lines mark π—π stacking interactions) into the active site of CDK1 (PDB ID: 4YC6).

The direct interaction of LCS1269 with CDK1 could dampen its function thus weakening the glioblastoma cells mitotic activity. The probable capability of LCS1269 to interact with CDK1 was evaluated by means of molecular modeling (Figure 7).

A docking analysis predicted that LCS1269 interacts with the CDK1 active center by means of five hydrogen bonds: two donor H-bonds between Asp10 and two hydroxyl groups at once, 3′-OH and 4′-OH, in sugar moiety were observed. Another donor H-bond between the Ser9 and 2′-OH group in the carbohydrate moiety was also modeled. The oxygen of the carbonyl group in the pyrrole ring and oxygen of amide group formed two acceptor H-bonds with Lys88 and Gln132 residues, respectively. Furthermore, the indole moiety of the indolocarbazole skeleton of LCS1269 formed two π—π stacking interactions with Trp168. The glide score of LCS1269 was −7.92, which was higher than that of the reference compound dinaciclib (−7.58) (Supplementary, Figure S2), thus indicating that LCS1269 ligand interactions with CDK1 target protein are probable.

### 2.5. CDK1 Modulation Mediated by LCS1269 May Be Associated with FOXM1/Plk1 Signaling and p21 Activation

Polo-like kinase Plk1 is another protein that is regulated in a cell cycle-dependent manner. Plk1 controls such processes as mitotic entry, centrosome separation and maturation, chromosome arm resolution, and microtubule–kinetochore attachment. Plk1 basal expression in interphase cells starts to increase at the completion of the S phase and reaches its maximum during the mitotic phase [51]. The inhibition of Plk1 leads to the arrest of cells in a prometaphase-like state. The phosphorylation of Thr210 is considered the first step triggering Plk1 activation during mitosis [52].

Plk1 inactivates both Wee1 and Myt1 by phosphorylation [53]. In our study, Plk1 expression, as well as Plk1 phosphorylated at Thr210, is stimulated in U87 and T98G glioblastoma cells due to the LCS1269 action as compared to the untreated cells, thus indicating that the inhibitory phosphorylation of Wee1 and Myt1 could be expected. However, Plk1 and p-Plk1 (Thr210) expression increased simultaneously with p-Wee1 (Ser642) and Myt1 expression (Figure 6, pink frame).

To phosphorylate Wee1 and Myt1 by Plk1, the previous CDK1-dependent phosphorylation of these CDK1 inhibitors is necessary. The phosphorylation of Wee1 at Ser123 and Myt1 on Thr478 by CDK1 creates a docking site for Plk1 phosphorylation [54]. CDK1 down-regulation, as well as its activated form, may be responsible for the decreased Wee1 and Myt1 phosphorylation by CDK1 and could thus prevent the inhibitory phosphorylation of these kinases by Plk1.

Plk1 phosphorylates at S715 and S724 FoxM1 (Forkhead box M1), a proliferation-associated transcription factor of the Forkhead box (Fox) protein superfamily, promoting its transcriptional activity [55]. The transcriptional activity of FoxM1 is upregulated through the cell cycle from a relative hypo-phosphorylation status in the G1/S phase to increased phosphorylation in the S phase and hyper-phosphorylation status in the M phase. FoxM1 stimulates the transcription of multiple G2/M-specific genes which are essential regulators of the mitotic entry, including *cyclin B*, *PLK1*, *Aurora B*, and *Cdc25* phosphatases [56]. Just as in the case of Wee1 and Myt1, FoxM1 needs the “prerequisite” phosphorylation by CDK1 providing a polo-box domain, which is necessary to be recruited and activated by Plk1 [57]. In our study, in spite of the elevation of FoxM1 expression in all glioblastoma cells due to the LCS1269 action, its transcriptional activity could also be reduced because of the decreased expression and functional activity of CDK1 (Figure 6, yellow frame).

Thus, the decrease in CDK1 expression and activity in consequence of LCS1269 action seems to be the key event leading to the G2 block of glioblastoma cells.

The passage of cells from G2 into mitosis could be arrested by the inhibitor proteins, such as p21 [47]. p21 associates with cyclin B1-Cdk1 complexes, and completely and irreversibly inactivates cyclin B1-Cdk1 complexes in the nucleus, thus rendering impossible their activation by Plk1 or Cdc25 phosphatases [58]. In our study, p21 expression was dose-dependently increased by LCS1269 treatment in U87 and U251 cells (Figure 6, orange frame), indicating that the augmentation of p21 expression could contribute to the G2/M cell cycle arrest. However, it should be mentioned that T98G cells were p21 negative. The expression of another cyclin-CDK inhibitor p27, structurally related to p21, was not changed by the action of LCS1269 in any of cells studied (Figure 6, orange frame).

### 2.6. In Silico Drug-Likeness and Absorption, Distribution, Metabolism, Excretion, and Toxicity (ADMET) Prediction

Drug-likeness is a complex balance of various molecular features of candidate drug, such as molecule size, hydrogen bonding characteristics, electronic distribution and hydrophobicity [59]. Only topological polar surface area (TPSA) of LCS1269 does not meet the acceptable value, while two and more physicochemical properties for reference compounds are found to exceed the optimal scores (Table 1). It should be noted that the TPSA metric was shown to be less important than the TPSA/NPSA (non-polar surface area) balance when analyzing potential drug candidates′ bioavailability [60].

However, the drug-likeness evaluation of LCS1269 based on the rule of thumb, such as Lipinski’s rule of five, does not assess this compound as a potential drug. On the other hand, only staurosporine and rebeccamycin, but not becatecarin, were shown to comply with Lipinski’s rule criteria.

Absorption and distribution refer to two important pharmacokinetic processes by which the unmetabolized drug can reach to the body circulation system and transfer from one tissue or organ to another [61]. From Table 2, it can be seen that the Caucasian colon adenocarcinoma cell line 2 (Caco—2) permeability score of LCS1269 (−5.70 log units) was indicated to be slightly higher than the minimum optimal score (−5.15 log units) and closely resembling those of the reference drugs. The Madin–Darby canine kidney (MDCK) permeability score of 5.0 × 10^−6^ cm/s corresponded a medium permeability (2—20.0 × 10^−6^ cm/s), which fell within the acceptable range. At the same time, the predicted values of human intestinal absorption (HIA) for all tested compounds, except becatecarin and LCS1269, did not exceed 30%. Only LCS1269 and becatecarin are anticipated to display a bioavailability greater than 30% (F_30%_ 0.213 and 0.011, respectively). The plasma protein binding (PPB) value of LCS1269 was comparable to those for reference drugs (optimal PPB < 90%). Additionally, the predicted outcomes for the blood–brain barrier (BBB) permeability of LCS1269 assumed a likelihood similar to that simulated for rebeccamycin and becatecarin. It is worth noting that the volume of distribution (VD) value determined for LCS1269 (0.75 L/kg) was slightly lower than that calculated for reference drugs. Nonetheless, all VD values fall within the optimal range (0.04–20.0 L/kg).

Metabolism plays a pivotal role in the biotransformation of exogenous substances to increase their water solubility so that they can be easily removed from the body by excretion [62]. The probability of the tested compound being an inhibitor of the five main isoenzymes (1A2, 2C19, 2C9, 2D6, and 3A4) of cytochrome P450 (CYP450) is one of the major causes leading to toxic or other unwanted adverse effects due to the lower clearance and accumulation of the drug or its metabolites [63]. As shown in Table 2, LCS1269 exhibited a weak inhibitory effect on CYP3A4 isoform, while reference drugs predicted an inhibitory ability against two (for rebecammycin and becatecarin) or three (for staurosporine) isoforms of CYP450.

The clearance rate (CL) of LCS1269 was evaluated to be 2.01 mL/min/kg, indicating a low range of clearance rate (<5 mL/min/kg). The half-life value (T_1/2_) for LCS1269 was calculated to be 0.16.

Besides good biological activity and favorable pharmacokinetic profile, it is also desirable to develop a novel drug that shows no or at least minor toxicity [64].

The toxicological behavior of LCS1269 in comparison with the reference drugs was determined by evaluating the predicted the inhibition of human ether-a-go-go related (hERG) gene expression (to check cardiovascular toxicity), human hepatotoxicity (H—HT), drug-induced liver injury (DILI), Ames’s test (to predict mutagenicity of the compounds) and a number of additional tests summarized in Table 3.

Overall, from Table 1, Table 2 and Table 3, it follows that most parameters of the ADMET profile for LCS1269 fall within the acceptable range, corresponding to or even surpassing similar ones for reference drugs. It justifies that LCS1269 exhibits good characteristics as a candidate molecule in the context of drug development for glioblastoma treatment.

## 3. Materials and Methods

### 3.1. Cell Lines and Cultures

The human glioblastoma multiforme (GBM) cell lines U87, U251, and T98G were purchased from the American Type Culture Collection (ATCC, Manassas, VA, USA). Three patient-derived primary cell cultures designated as Gbl1 (G01), Gbl2 (G22), and Gbl3 (Sus\fP2) were obtained from human GBM tissues after tumor resection complying with all formal requirements of the Russian Federation and in accordance with the Declaration of Helsinki as previously described [65,66,67]. This study was approved by the Ethics Committee of N.N. Burdenko National Medical Research Center of Neurosurgery, the Ministry of Health of the Russian Federation (№_12/2020 dated 15 December 2020). All investigated cells were cultured in high-glucose DMEM medium (Paneco, Moscow, Russia) supplemented with 1% L-glutamine (Paneco, Moscow, Russia), 10% fetal bovine serum (Biosera, Cholet, France), 100 IU/mL penicillin, and 100 μg/mL streptomycin at 37 °C with a humidified atmosphere of 5% CO_2_ incubator.

### 3.2. Cell Viability Assay

To investigate cytotoxic activity of LCS1269 and temozolomide (Sigma-Aldrich, Saint Louis, MO, USA) as a reference drug, standard MTT assay was performed as previously described [34]. Briefly, cells (5000 per well) were plated in 96-well culture plates and cultivated for 16–18 h to be attached. Then, LCS1269 or temozolomide was added to cells with the final concentrations 0.39–50 μmol/L (serial dilution 1:1) for LCS1269 and 25–1000 μmol/L for temozolomide, respectively, and incubated at 37 °C in a 5% CO_2_ incubator for three days. After three days, a 20 μL MTT solution (5 mg/mL) was added to each well, followed by incubation for three hours. After this, 60 μL DMSO (Paneco, Moscow, Russia) was added to dissolve formazan crystals. The absorbance was measured at a wavelength of 540 nm in a microplate reader Multiskan FC (ThermoScientific, Waltham, MA, USA). The half-maximal concentration at which 50% of cells were viable as compared to the control was calculated as IC50 in μmol/L. Each experiment was performed in triplicate to calculate the average values and repeated at least three times.

### 3.3. Cell Cycle Analysis

The U87, U251, and T98G cells were seeded into 6-well plates (2 × 10^5^ cells per well) in complete culture medium. Cell cycle analysis was performed as previously described [34]. Briefly, the cells were treated with the different concentrations of LCS1269 for 24 h. The attached cells were rinsed two times with PBS and collected using a 0.25% trypsin-EDTA solution (Paneco, Moscow, Russia). Next, 70% ethanol was used for cell fixation (4 °C, at least 3 h). After fixation, the cells were centrifuged (1000 rpm, 5 min) at room temperature, then washed once with PBS, and 10 μL of RNAse A (10 mg/mL) for 30 min at 37 °C was added. After the suspending of cell pellets in 400 μL of PI solution (0.5 mg/mL in PBS) followed by incubation for 30 min in the dark, the DNA content was analyzed using a FACSCanto2 flow cytometer (BD Biosciences, Franklin Lakes, NJ, USA). The fluorescence emitted from the PI–DNA complex was estimated at 488 nm using at least 10,000 cells per sample. The ModFit LT v. 3.2 software package (Verity Software House, Topsham, ME, USA) was used to determine the distribution of cells in the G0/G1, S, and G2/M phases.

### 3.4. Hoechst 33258 Staining

The U87, U251, and T98G cells were seeded on coverslips in a 6-well culture plate and grown overnight. The next day, cells were exposed with 0.1% DMSO (untreated) or 2.5 μM LCS1269 for 24 h. Following treatment, cells were rinsed and fixed with 4% paraformaldehyde in PBS for 30 min. Then, the cells were stained with Hoechst 33258 (Sigma-Aldrich, Saint Louis, MO, USA) dye solution (10 μg/mL) for 20 min and imaged in random fields at a magnification of 630× using Axioplan 2 fluorescence microscope and AxioVision v. 4.1 software (Carl Zeiss AG, Oberkochen, Germany).

### 3.5. RNA Extraction, Reverse Transcription, and Quantitative Real-Time PCR

All procedures were previously described [34]. Briefly, the total RNA was extracted from cells using TRI reagent (Molecular Research Center, Cincinnati, OH, USA). The reverse transcription PCR reactions were performed with 1 μg of total RNA using oligo-(dT)_15_ primer (Syntol, Moscow, Russia) and M-MuLV reverse transcriptase. The assays were carried out using EvaGreen dye (BIOTIUM, Fremont, CA, USA) on a CFX96 real-time PCR detection system (Bio-Rad, Hercules, CA, USA, USA). PCR conditions were the same for all genes: preheating at 95 °C, 5 min, followed by 39 cycles of 95 °C for 20 s, 59 °C for 25 s, and 72 °C for 20 s. The tyrosine 3-monooxygenase/tryptophan 5-monooxygenase activation protein zeta (YWHAZ) gene was used as the internal control for RNA template normalization and data standardization. The primers used for amplification were as follows: Aurora-B, 5′-GACACCCGACATCTTAACGC-3′ (forward) and 5′-CGCCCTCCTTCTCTATCTGG-3′ (reverse); YWHAZ, 5′-ACTTTTGGTACATTGTGGCTTCAA-3′ (forward) and 5′-CCGCCAGGACAAACCAGTAT-3′ (reverse). The relative gene expression level was calculated using the 2^–∆∆Ct^ equation (∆Ct = Ct (YWHAZ) − Ct (test gene)), where Ct is the threshold cycle of the gene in the exponential phase of the amplification curve.

### 3.6. Western Blotting

The cells were lysed with RIPA buffer (ThermoScientific, Waltham, MA, USA). The whole protein extracts were prepared, and the protein concentration was measured using a BCA protein assay kit (Millipore, South San Francisco, CA, USA). Equal amounts (30 μg) of protein samples were separated by SDS-PAGE and transferred onto a nitrocellulose membrane using a trans-blot system (Bio-Rad, USA). After the blocking of non-specific protein binding, blots were incubated at 4 °C overnight with specific primary antibodies (Supplementary Table S1). Further, the blots were washed with TBS/Tween-20 and incubated at room temperature for 1 h with horseradish peroxidase (HRP)-conjugated secondary antibodies (Jackson ImmunoResearch, West Grove, PA, USA). After washed in TBS/Tween-20, protein bands were visualized with enhanced chemiluminescence (ECL) substrate (ThermoScientific, Waltham, MA, USA) and imaged by Image Quant LAS4000 (GE HealthCare, Chicago, IL, USA). The β-Actin protein level served as a loading control. The publicly available ImageJ v. 2.14.0 software program (National Institutes of Health, Bethesda, MD, USA) was applied to measure the expression of investigated proteins.

### 3.7. In Vivo Xenograft Growth Experiments

To estimate the anti-tumor potency delivered by LCS1269 in vivo, ten nude mice (male BALB/c immunodeficient mice, 7–8 weeks of age) were used following the standard protocol [34]. U87 cells, demonstrating tumorigenic potential, were subcutaneously injected (10^7^ cells per animal) to induce xenograft formation [https://www.atcc.org/products/htb-14, accessed on 5 November 2024]. LCS1269 (60 mg/kg) was administered intraperitoneally to the treated group of mice (*n* = 5) 48 h after inoculation, every 24 h for five days, while the control group of mice (*n* = 5) was injected intraperitoneally with the physiological solution under the same conditions. To evaluate the tumor growth in treated with LCS1269 and control groups of U87-bearing nude mice the tumor volume was calculated using tumor length × width^2^/2 equation. The body weight of mice was monitored every 3 days. All experimental procedures and protocols were performed in accordance with the National Institutes of Health Guidelines for the Care and Use of Laboratory Animals and approved by Animal Ethics Committee of N.N. Blokhin National Medical Research Center of Oncology the Ministry of Health of the Russian Federation (№ 01c/p-2024 on 4 June 2024).

### 3.8. Molecular Docking

Aurora B in complex with inner centromere protein (4AF3) and CDK1 (4YC6) were selected as target proteins for molecular modeling. The structures were taken from the open database PDB (Protein Data Bank). Molecular modeling was performed in Maestro 11 software package (Schrödinger Inc., Portland, OR, USA). The first stage of preparation was performed using the “Protein Preparation Wizard” panel and included protonation when pH = 7.0, the removal of water molecules and other solvents, and the restoration of disulfide bridges and loops in protein structures. Next, a single minimization was performed using a force field, OPLS3. The binding sites of the target proteins with the ligand were analyzed in the Sitemap program included in the Maestro 11 software package. The procedure of the ligand preparation included graphical representation, protonation when pH = 7.0, and the determination of conformers by “LigPrep” panel Maestro 11 program. To localize the ligand binding site in the active center of the target protein, a 20–30 Å box was superimposed on the region of the putative active center using the “Receptor grid generation” panel. The Maestro 11 platform standard Glide docking protocol was used to quantify interactions between target proteins and ligands.

### 3.9. Drug-Likeness and ADMET Studies

The drug-likeness and ADMET descriptors of LCS1269 with staurosporine, rebeccamycin, and becatecarin serving as reference drugs was in silico predicted utilizing ADMETlab 2.0 (https://admetmesh.scbdd.com, accessed on 5 November 2024) [68].

### 3.10. Statistical Analysis

The data obtained were presented as the mean ± SD and analyzed with GraphPad Prism v. 5.02 software (GraphPad Software, San Diego, CA, USA). All experiments described in the present article were independently repeated at least three times. Statistical comparisons were carried out by a one-way analysis of variance (ANOVA) following by a Newman–Keuls multiple comparison test or Student’s *t*-test. Statistical significance was defined as * *p* < 0.05, ** *p* < 0.01, *** *p* < 0.001.

## 4. Conclusions

We demonstrated that LCS1269 prominently suppressed the viability of both glioblastoma cell lines and patient-derived glioblastoma cultures. This compound exhibited significantly more profound growth-inhibiting effect as compared to temozolomide. We revealed that LCS1269 arrested glioblastoma cells in the G2 period interphase hindering the mitosis entry. The molecular docking simulations identified CDK1 as a potential target for LCS1269 inhibitory activity. Importantly, our mechanistic studies further confirmed that LCS1269 may also affect CDK1 activity through the regulation of Wee1/Myt1 signaling and FOXM1/Plk1 signaling pathways, as well as p21 up-regulation. In addition, a U87-bearing xenograft model obviously demonstrated that LCS1269 induced the profound growth delay in vivo. Finally, ADMET evaluation predicted that LCS1269 had some suitable pharmacological and toxicological properties. In summary, our findings clearly characterize LCS1269 as a potential compound for anti-glioblastoma therapy that warrants further investigation.

## Figures and Tables

**Table 1:** Physiochemical properties for LCS1269, staurosporine, rebeccamycin, and becatecarin.

Compound	MW	nHA	nHD	TPSA	Log *P*	Log *D*	Lipinski’s Rule
**LCS1269**	579.18	12	5	170.01	1.89	2.04	No
**Staurosporine**	466.20	7	2	69.45	4.93	3.59	Yes
**Rebeccamycin**	569.08	10	5	146.04	3.22	2.83	Yes
**Becatecarin**	668.18	11	4	140.49	4.62	3.39	No
**Optimal value**	≤600	≤12	≤7	≤140	≤3	1–3	

MW: molecular weight; nHA: number of hydrogen acceptors; nHD: number of hydrogen donors; TPSA: topological polar surface area; Log *P*: log of the octanol/water partition coefficient; Log *D*: log P at physiological pH 7.4; Lipinski’s rule: MW ≤ 500, log *P* ≤ 5; nHA ≤ 10, nHD ≤ 5.

**Table 2:** Pharmacokinetic profiles assessment of LCS1269, staurosporine, rebeccamycin, and becatecarin.

Compound	Absorption				Distribution			Metabolism					Excretion	
	Caco-2, log Unit	MDCK, ×10^−6^ cm/s	HIA	F_30%_	PPB, %	BBB	VD, L/kg	CYP1A2 Inhibitor	CYP2C19 Inhibitor	CYP2C9 Inhibitor	CYP2D6 Inhibitor	CYP3A4 Inhibitor	CL mL/min/kg	T_1/2_, a.u.
**LCS1269**	−5.70	5.0	++	− −	98.06	− − −	0.75	− − −	− −	−	− − −	+	2.01	0.16
**Staurosporine**	−5.62	5.0	++	+++	95.04	+	1.06	+	−	++	−	++	8.56	0.04
**Rebeccamycin**	−5.73	5.0	+++	+++	96.15	− − −	1.16	−	− −	+	+	− −	2.24	0.08
**Becatecarin**	−5.64	9.0	− −	− − −	94.11	− − −	1.08	+	− −	++	−	−	6.53	0.04

Caco-2: Caucasian colon adenocarcinoma cell line 2; MDCK: Madin–Darby canine kidney; HIA: human intestinal absorption; F30%: 30% bioavailability; PPB: plasma protein binding; BBB: blood–brain barrier penetration; VD: volume of distribution; CYP: cytochrome P450; CL: clearance; T_1/2_: time of half-life (a.u.—arbitrary units). For endpoints classification, the prediction probability values are transformed in six symbols: 0–0.1 (− − −), 0.1–0.3 (− −), 0.3–0.5 (−), 0.5–0.7 (+), 0.7–0.9 (++), and 0.9–1.0 (+++).

**Table 3:** Toxicity prediction for LCS1269, staurosporine, rebeccamycin, and becatecarin.

Compound	hERG Blockers	H−HT	DILI	Ames Mutagenicity	AROT	FDAMDD	SS	Carcinogenicity
**LCS1269**	− − −	− −	+++	− −	++	++	− − −	++
**Staurosporine**	++	++	−	++	−	+++	− − −	+++
**Rebeccamycin**	− − −	+	+++	−	− −	++	−	++
**Becatecarin**	++	++	+++	−	− − −	++	− − −	− − −

hERG: human ether-a-go-go-related gene (cardiac toxicity); H—HT: human hepatotoxicity; DILI: drug-induced liver injury; AROT: acute rat oral toxicity; FDAMDD: FDA (Food and Drug Administration) maximum recommended daily dose; SS: skin sensitization. For empirical decisions, the prediction probability values are transformed in six symbols: 0–0.3 (− − −), 0.1–0.3 (− −), 0.3–0.5 (−), 0.5–0.7 (+), 0.7–0.9 (++), and 0.9–1.0 (+++).

## Data Availability

The original contributions presented in this study are included in the article/Supplementary Material. Further inquiries can be directed to the corresponding author.

## Supplementary Materials

The following supporting information can be downloaded at: https://www.mdpi.com/article/10.3390/ph17121642/s1, Figure S1: Ligand–protein interaction modeling based on the molecular docking of LCS1269 with active site of Aurora B in complex with inner centromere protein (PDB ID: 4AF3); Figure S2: Ligand–protein interaction modeling based on the molecular docking of dinaciclib with active site of CDK1 (PDB ID: 4YC6); Table S1: Primary antibodies used in Western blot analyses; Figure S3: HPLC analysis of LCS1269.
