# Supplementary material for: N-Glycoside of Indolo[2,3-a]pyrrolo[3,4-c]carbazole LCS1269 Exerts Anti-Glioblastoma Effects by G2 Cell Cycle Arrest and CDK1 Activity Modulation: Molecular Docking Studies, Biological Investigations, and ADMET Prediction
Source: Pharmaceuticals (Basel). 2024 Dec 6;17(12):1642. doi: 10.3390/ph17121642 (PMC11676706; doi:10.3390/ph17121642)
Supplement: Supplementary file 1 [file pharmaceuticals-17-01642-s001.zip › pharmaceuticals-3328791-supplementary.pdf]

N-glycoside of indolo[2,3-a]pyrrolo[3,4-c]carbazole LCS1269 exerts anti-glioblastoma effects by G2 cell cycle arrest and CDK1 activity modulation: molecular docking studies, biological investigations, and ADMET prediction

Nikolay Kalitin [1], Natalia Koroleva [2], Anna Lushnikova [2], Maria Babaeva [3], Nadezhda Samoylenkova [4], Ekaterina Savchenko [4], Galina Smirnova [5], Yulia Borisova [5], Alexander Kostarev [6], Aida Karamysheva [1] and Galina Pavlova [4, 7]

[1] *Laboratory of Tumor Cell Genetics, N.N. Blokhin National Medical Research Center of Oncology, 115478 Moscow, Russia*

[2] *Laboratory of Oncogenomics, N.N. Blokhin National Medical Research Center of Oncology, 115478 Moscow, Russia*

[3] *Master's in Molecular Medicine, Charité – Universitätsmedizin Berlin, 10117 Berlin, Germany*

[4] *Laboratory of Molecular and Cellular Neurogenetics, N.N. Burdenko National Medical Research Center of Neurosurgery, 125047 Moscow, Russia*

[5] *Laboratory of Biochemical Pharmacology and Tumor Models, N.N. Blokhin National Medical Research Center of Oncology, 115478 Moscow, Russia*

[6] *Max Planck Institute for Biology, University of Tübingen, 72074 Tübingen, Germany*

[7] *Laboratory of Neurogenetics and Developmental Genetics, Institute of Higher Nervous Activity and Neurophysiology of RAS, 117485 Moscow, Russia*

Corresponding author:

Nikolay Kalitin, PhD, N.N. Blokhin National Medical Research Center of Oncology, 24 Kashirskoe Shosse, 115478 Moscow, Russia. E-mail: f.oskolov@mail.ru

A

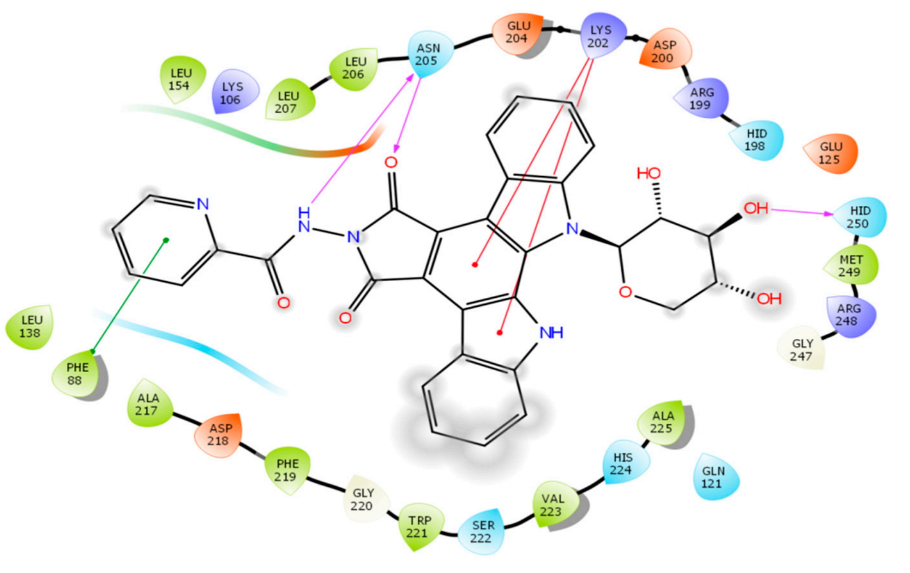

B

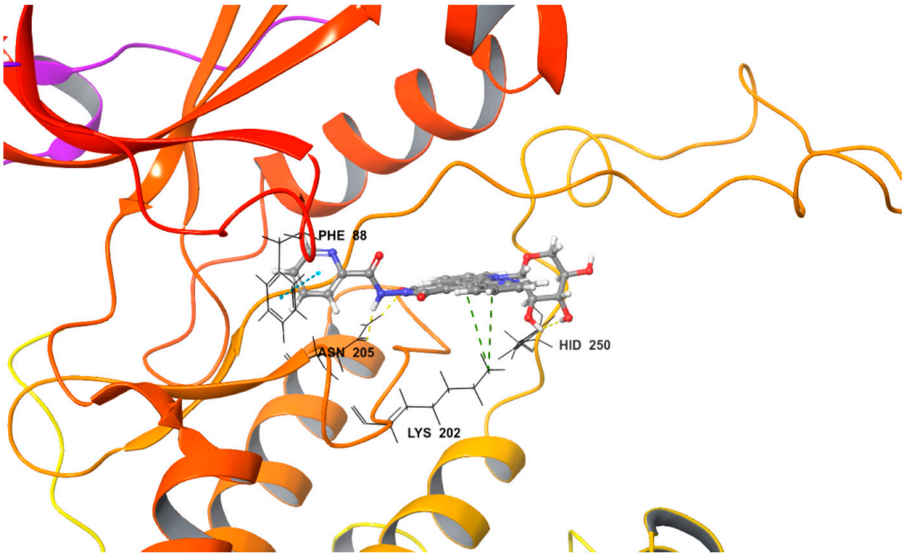

Figure S1. Ligand-protein interaction modelling based on the molecular docking of LCS1269 with active site of Aurora B in complex with inner centromere protein (PDB ID: 4AF3). (A) 2D binding mode (purple arrows denote hydrogen bonds, red lines represent cation – π interactions and green line marks π–π stacking interaction). (B) 3D binding mode (green dashed lines show cation–π interactions, blue dashed line indicates π – π stacking interaction and yellow dashed lines illustrate hydrogen bonds)

A

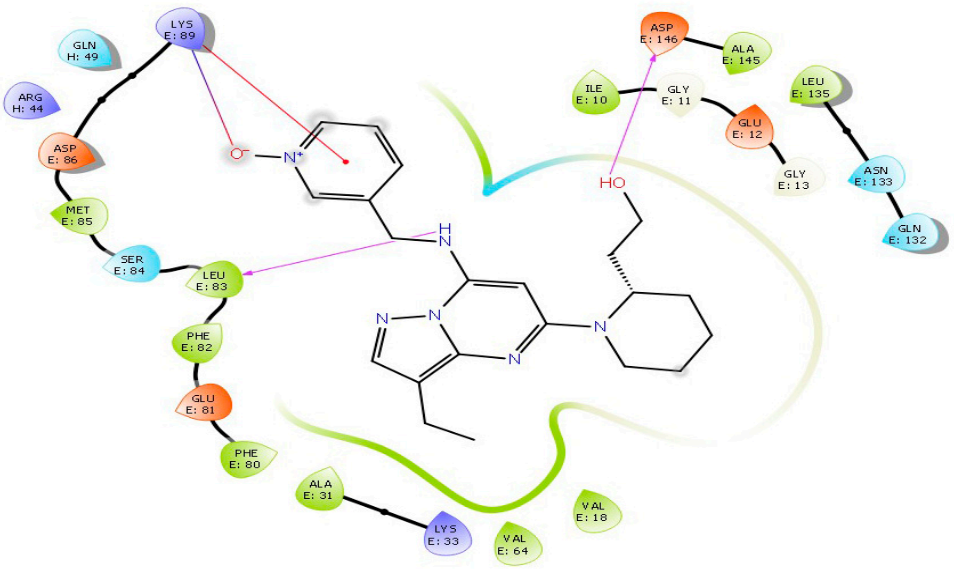

B

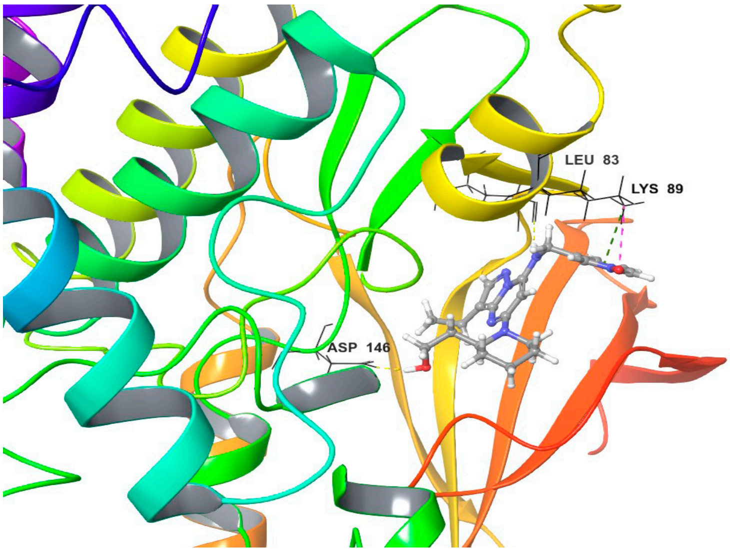

Figure S2. Ligand-protein interaction modelling based on the molecular docking of dinaciclib with active site of CDK1 (PDB ID: 4YC6). (A) 2D binding mode (purple arrows denote hydrogen bonds, red line represents cation – π interaction and red-blue line marks salt bridge). (B) 3D binding mode (green dashed lines shows cation – π interaction, pink dashed line indicates salt bridge and yellow dashed lines illustrate hydrogen bonds)

Table S1. Primary antibodies used in Western blot analyses

Antibody name	Manufacturer	Catalogue number	Dilution rate
p-Histone H3 (Ser10) (D2C8) XP® Rabbit mAb	Cell Signaling Technology	3377	1:1000
CDK1 (POH1) Mouse mAb	Cell Signaling Technology	9116	1:1000
p-CDK1 (Tyr15) (10A11) Rabbit mAb	Cell Signaling Technology	4539	1:1000
Cyclin B1 (D5C10) XP® Rabbit mAb	Cell Signaling Technology	12231	1:1000
Myt1 Antibody	Cell Signaling Technology	4282	1:1000
p-Wee1 (Ser642) (D47G5) Rabbit mAb	Cell Signaling Technology	4910	1:1000
Cdc25C (5H9) Rabbit mAb	Cell Signaling Technology	4688	1:1000
p-Cdc25C (Ser216) (63F9) Rabbit mAb	Cell Signaling Technology	4901	1:1000
p21 Waf1/Cip1 (12D1) Rabbit mAb	Cell Signaling Technology	2947	1:1000
p27 Kip1 (D69C12) XP® Rabbit mAb	Cell Signaling Technology	3686	1:1000
FoxM1 (D12D5) XP® Rabbit mAb	Cell Signaling Technology	5436	1:1000
PLK1 (208G4) Rabbit mAb	Cell Signaling Technology	4513	1:1000
p-PLK1 (Thr210) Antibody	Cell Signaling Technology	5472	1:1000
beta-Actin (C4) Mouse mAb HRP	Santa Cruz Biotechnology	sc-47778 HRP	1:500

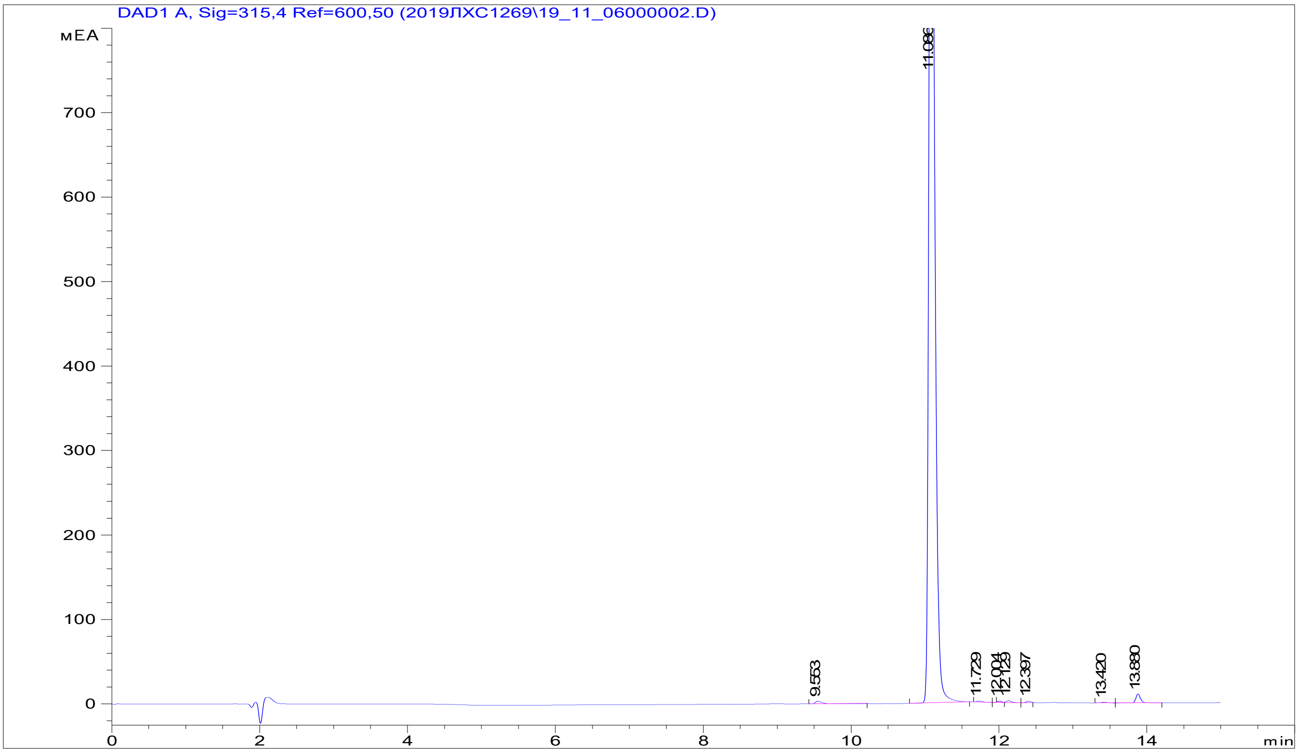

Figure S3. HPLC analysis of LCS1269. LCS1269 purity was 98 – 99%. The gradient elution at 40°C was carried out. The gradient started at water/acetonitrile (9:1) + trifluoroacetic acid 0.1% (mobile phase A) and ended at water/acetonitrile (0.5:9.5) + trifluoroacetic acid 0.1% (mobile phase B) after 15 min. The retention time was 10.9 ± 0.2 min, λ 315 nm.
